# Role of Hydrogen Sulfide in NRF2- and Sirtuin-Dependent Maintenance of Cellular Redox Balance

**DOI:** 10.3390/antiox7100129

**Published:** 2018-09-28

**Authors:** Tiziana Corsello, Narayana Komaravelli, Antonella Casola

**Affiliations:** 1 Department of Pediatrics, University of Texas Medical Branch at Galveston, Galveston, TX 77555, USA; ticorsel@utmb.edu (T.C.); nakomara@utmb.edu (N.K.); 2 Department of Microbiology and Immunology, University of Texas Medical Branch at Galveston, Galveston, TX 77555, USA

**Keywords:** hydrogen sulfide, oxidative stress, redox, *NRF2*, sirtuin

## Abstract

Hydrogen sulfide (H_2_S) has arisen as a critical gasotransmitter signaling molecule modulating cellular biological events related to health and diseases in heart, brain, liver, vascular systems and immune response. Three enzymes mediate the endogenous production of H_2_S: cystathione β-synthase (*CBS*), cystathione γ-lyase (*CSE*) and 3-mercaptopyruvate sulfurtransferase (*3-MST*). *CBS* and *CSE* localizations are organ-specific. *3-MST* is a mitochondrial and cytosolic enzyme. The generation of H_2_S is firmly regulated by these enzymes under normal physiological conditions. Recent studies have highlighted the role of H_2_S in cellular redox homeostasis, as it displays significant antioxidant properties. H_2_S exerts antioxidant effects through several mechanisms, such as quenching reactive oxygen species (ROS) and reactive nitrogen species (RNS), by modulating cellular levels of glutathione (GSH) and thioredoxin (*Trx-1*) or increasing expression of antioxidant enzymes (AOE), by activating the transcription factor nuclear factor (erythroid-derived 2)-like 2 (*NRF2*). H_2_S also influences the activity of the histone deacetylase protein family of sirtuins, which plays an important role in inhibiting oxidative stress in cardiomyocytes and during the aging process by modulating AOE gene expression. This review focuses on the role of H_2_S in *NRF2* and sirtuin signaling pathways as they are related to cellular redox homeostasis.

## 1. Introduction

**Figure 1:**
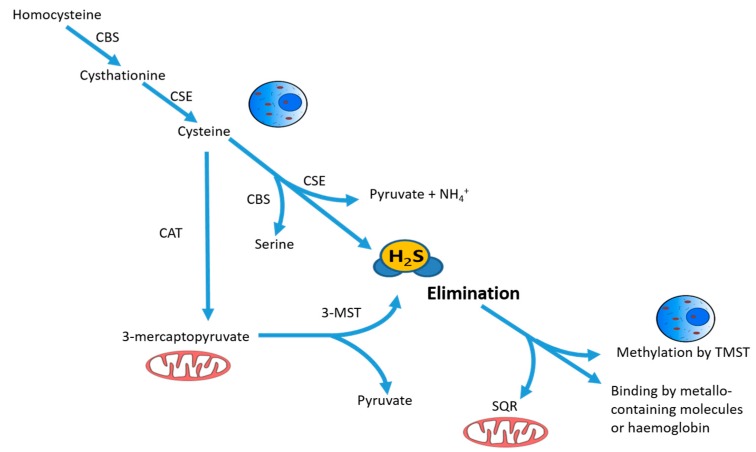
Schematic description of intracellular synthesis and degradation of hydrogen sulfide H_2_S. H_2_S is produced by cytoplasmic and mitochondrial enzymes cystathionine γ-lyase (*CSE*), cystathionine β-synthase (*CBS*), 3-mercaptopyruvate sulfurtransferase (*3-MST*) and cysteine aminotransferase (*CAT*) using cysteine or homocysteine as substrates. The intracellular non-toxic H_2_S level is being actively maintained by oxidation in mitochondria by the enzyme sulfide:quinone reductase (SQR), together with rhodanese and sulfur dioxygenase, or by methylation in the cytoplasm using thiol S-methyltransferase (TMST). Free H_2_S can also be bound by methemoglobin and by molecules with metallic or disulfide bonds and excreted with biological fluids. Reprinted with permission of the American Thoracic Society. Copyright © 2018 American Thoracic Society [14].

Hydrogen sulfide (H_2_S) is an inorganic and colorless gas, with strong odor and toxic effects at high concentrations [1]. In the last few years, H_2_S has been identified as the third most physiologically important gasotransmitter participating in multiple cellular signaling pathways, along with carbon monoxide (CO) and nitric oxide (NO) [2]. It plays a physiological role in a variety of cellular and organ functions and a protective role in multiple pathological conditions, displaying vasoactive, cytoprotective, anti-inflammatory and antioxidant activities (reviewed in [3]). As a gasotransmitter, it diffuses quickly through the cells, operating next to sites of biosynthesis, with a short lifetime [4]. Endogenous H_2_S in mammals is generated through enzymatic and non-enzymatic pathways. The former process requires the action of cytosolic and mitochondrial enzymes: cystathionine β-synthase (*CBS*), cystathionine γ-lsase (*CSE*), 3-mercaptopyruvate sulfurtransferase (*3-MST*) and cysteine aminotransferase (CAT), using L-cysteine or homocysteine as substrates (Figure 1). *CBS* and *CSE* are mainly expressed in the vascular, nervous, and cardiovascular systems, as well as in the liver and kidney [5,6]. These two enzymes are primarily responsible for H_2_S production, and they are also released in the circulatory system by hepatocytes and endothelial cells, as a part of the plasma [7]. The non-enzymatic pathway is based on the reduction of sulfur species and thiol molecules, contributing in a minor extent to H_2_S cellular content.

Three distinct mechanisms are implicated in the catabolism of H_2_S: (1) oxidation, (2) methylation, and (3) scavenging by metalloproteins. Oxidation is the most common reaction, and encompasses the rapid metabolism of H_2_S to sulfate and sulfite species with thiosulfate as an intermediate molecule. It takes place in the mitochondria through the sequential action of sulfide: quinone oxidoreductase (SQR), rhodanese and sulfur dioxygenase. Methylation converts endogenous H_2_S into dimethylsulfide and thiol S-methyltransferase (TMST) mainly in the cytoplasm, and it seems to have a lesser role than the oxidation pathway. Scavenging by metalloproteins involves the binding of H_2_S and hemoglobin by scavenging reaction, producing disulfide or metallo-containing products [8].

Free H_2_S exists in equilibrium with a pool of labile sulfur-containing molecules that can release H_2_S under certain physiological conditions. It has become more and more evident that part of the signaling effects attributed to H_2_S result from the occurrence of persulfides and polysulfides, among other sulfur-containing molecules, which have been collectively termed as “reactive sulfur species” (RSS). For more details on H_2_S metabolism and the formation of persulfides and polysulfides, as well as their role in cellular functions and signaling, please refer to the numerous excellent recent reviews published, such as [9,10,11,12,13].

Several studies have highlighted the role of H_2_S/RSS in cellular redox homeostasis, which occurs in part by modulating levels of cellular antioxidants, such as gluthatione (GSH), and increasing expression of antioxidant enzymes (AOE), and increasing activities/expressions of the transcription factor nuclear factor (erythroid-derived 2)-like 2 (*NRF2*) and the histone deacetylase protein family of sirtuins (*SIRTs*). This review summarizes the known role of H_2_S in maintaining cellular redox balance through these two mechanism(s) and its relationship with oxidative stress-related diseases.

## 2. Oxidative Stress and Antioxidant Effects of Hydrogen Sulfide

**Figure 2:**
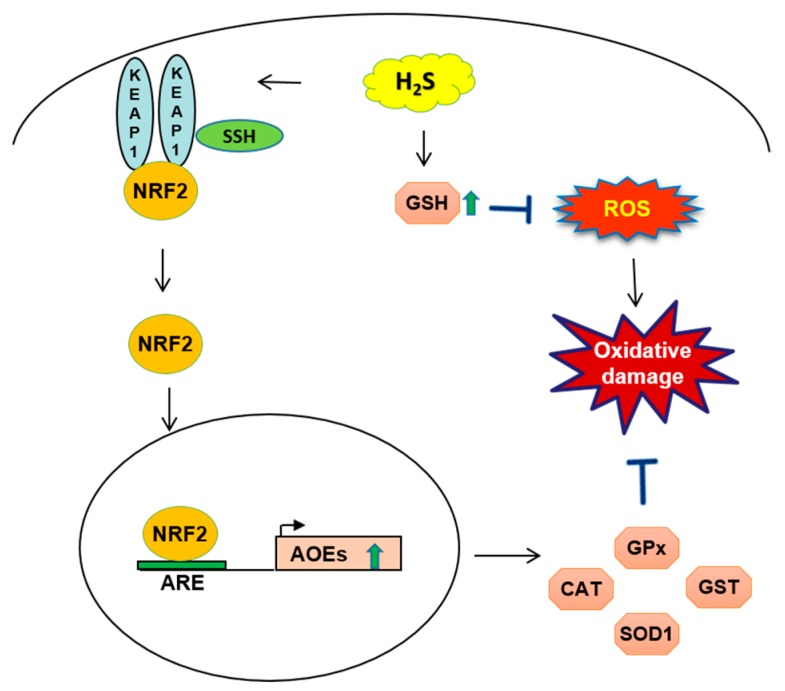
Schematic of H_2_S mechanism related to glutathione GSH and nuclear factor (erythroid-derived 2)-like 2 *NRF2* targets in oxidative cell-damage. The endogenous release of H_2_S increases GSH synthesis and blocks reactive oxygen species ROS production. When the cellular level of H_2_S is increased, Kelch-like ECH-associated protein 1 *Keap1* protein is S-sulfhydrated SSH: which brings a conformational change of the protein and *NRF2* release from *Keap1*. *NRF2* translocates to the nucleus, binding to the promoter containing antioxidant response element (ARE) sequences and increased transcription of antioxidant genes as catalase *CAT*, superoxide dismutase *SOD1*, glutathione-S-transferase *GST*, glutathione peroxidase *GPx*. AOE: antioxidant enzyme.

Reactive oxygen species (ROS) are ubiquitous, highly reactive molecules produced as a result of the reduction of molecular oxygen. Cellular sites for ROS generation include the mitochondria, and microsomes and require the involvement of various enzymes like cyclooxygenase, lipoxygenase, xanthine oxidase and membrane-bound reduced nicotinamide adenine dinucleotide phosphate NADPH-oxidase. Excessive levels of ROS can be generated by increased stimulation of the NADPH-oxidase system (mitochondrial and cell membrane-associated) or by other mechanisms, often involving mitochondrial dysfunction. Oxidative stress represents an imbalance between the ROS generation and the cellular antioxidant defensive system, which includes scavenging and repairing molecules. The first include a number of AOEs, such as superoxide dismutase (*SOD*) (three isoforms of *SOD* have been identified in mammals: the cytoplasmic *Cu/ZnSOD* or *SOD1*, the mitochondrial *MnSOD* or *SOD2*, and the extracellular *ECSOD* or *SOD3*), catalase and glutathione peroxidase (*GPx*). The latter include glutathione (GSH) and thioredoxin (*Trx-1*), which are the predominant antioxidants acting as a defense net during the oxidative stress process [15,16]. GSH is a tripeptide made of cysteine, glycine and glutamate, existing often as a reduced form, and it is synthesized from cysteine. GSH reduces disulfide bonds formed within cytoplasmic proteins to cysteines by serving as an electron donor. In the process, GSH is converted to its oxidized form, glutathione disulfide (GSSG). *Trx-1* is a 12-kD oxidoreductase enzyme containing a dithiol–disulfide active site, which acts as an antioxidant by facilitating the reduction of other proteins by cysteine thiol-disulfide [17]. H_2_S has been shown to exert antioxidant effects through several mechanisms including direct quenching of ROS, modulation of cellular levels of GSH and *Trx-1*, or increased expression of AOE, by activating the transcription factor nuclear factor (erythroid-derived 2)-like 2 (*NRF2*), as described below and summarized in Figure 2.

### 2.1. H_2_S and Repairing Antioxidant Defenses

H_2_S has been shown to be able to scavenge ROS and reactive nitrogen species (RNS), including hypochlorous acid, hydrogen peroxide, lipid hydroperoxides, superoxide and peroxynitrite (reviewed in [18,19]). Molecules containing an SH group such as H_2_S, HS–, HS–SH, and HSS– can reverse the damage due to ROS/RNS by donating a hydrogen atom to carbon-centered radicals; however, the very low concentrations of H_2_S and related molecules in blood and tissues limit their efficacy of repairing free radical cellular damage.

Cysteine, in addition to be a precursor for H_2_S, is also the source of GSH production. Cysteine exists as two unstable redox forms in the body: the oxidized form—cystine and the reduced form—cysteine. The extracellular cystine form is carried into cells through the cystine/glutamate antiporter system, after which cysteine is reduced and ready for GSH synthesis. The release of H_2_S into the extracellular space has been shown to induce a reduction of cystine into cysteine, increasing the amount of cysteine available as a substrate for GSH synthesis, and to enhance cystine transport [20]. GSH is synthesized by the consecutive catalysis of two enzymes, γ-glutamyl cysteine synthetase (*γ-GCS*) and glutathione synthetase (*GS*). H_2_S administration has been shown to enhance *γ-GCS* activity, without changing its expression [20]. H_2_S administration is also associated with augmented levels of GSH in the mitochondria. As cytoplasmic GSH is transported into mitochondria, because mitochondria cannot synthesize GSH, the enhanced mitochondrial GSH concentration following H_2_S administration is suggested to depend on the increased cytoplasmic GSH levels and enhanced transport into the mitochondria [20].

As mentioned above, thioredoxins are small thiol-oxidoreductase enzymes that control cellular redox homeostasis. In a mouse model of ischemia-induced heart failure, H_2_S treatment increased the *Trx-1* gene and protein levels, as well as basal *Trx-1* activity. H_2_S-dependent cardioprotection was dependent on an intact *Trx-1* protein [21]. H_2_S has been found to up-regulate *Trx-1* in part through an *NRF2*-independent, unidentified mechanism [22].

### 2.2. H_2_S-Mediated NRF2 Activation

*NRF2* is a basic leucine-zipper protein, belonging to the Cap’n’Collar family of transcription factors, that mediates expression of cytoprotective genes that defend cells from oxidative stress and cellular damage, including AOEs. *NRF2*-driven gene expression occurs through *NRF2* binding to promoters’ antioxidant responsive element (ARE) sequences. Under normal physiologic conditions, this transcription factor is confined to the cytoplasm by binding to Kelch-like ECH-associated protein 1 (*Keap1*) dimer forming an inactive complex. Whenever a change in redox status occurs by increased cellular ROS levels, *Keap1* dimer changes conformation due to the breaking of disulfide bonds between cysteine residues, and releases *NRF2*, which translocates to the nucleus and induces the transcription of AOE genes to attain redox homestastis [23].

At pH 7.4 under normal conditions, H_2_S is present mainly as dissociated anion (HS^−^, S^2−^) and 20% as not dissociated species. S-sulfhydration or persulfidation is a post-translational modification in which a sulfhydryl group (R-SH) attaches to the cysteine residues of target proteins in order to regulate the protein function. A variety of key proteins acting as a switch/sensor of different cellular pathways in mammals are sulfhydrated by H_2_S, leading to modulation of cell signaling that relates to oxidative stress, cell survival/death, metabolism, cell proliferation, and inflammation [24,25]. Various studies have shown that S-sulfhydration is one mechanism where H_2_S interacts directly with the *NRF2* pathway. H_2_S has been shown to S-sulfhydrate *Keap1* at the cysteine-151 residue, leading to *NRF2* dissociation, increased nuclear translocation and expression of antioxidant genes through binding to promoters’ ARE sites [26]. Furthermore, H_2_S can S-sulfhydrate *Keap1* at the cysteine-226 and cysteine-613 residues, leading to *Keap1* inactivation, *NRF2* release and promotion of *NRF2*-dependent gene expression [27].

## 3. H_2_S and Sirtuin Interaction during Oxidative Stress

*SIRTs* are enzymes that catalyze post-translational modifications of both histone and nonhistone proteins. There are seven members in the mammalian family with different cellular localizations, enzymatic activities and targets (reviewed in [28]). *SIRT1* and *SIRT6* are present in the nucleus; *SIRT2* is in the cytoplasm; *SIRT3*, *SIRT4*, *SIRT5* are localized in the mitochondria. Originally identified as deacetylases, *SIRTs* have more recently been found to catalyze a variety of other reactions, including desuccinylation, demalonylation, and deglutarylation. *SIRTs* are classified as class III histone deacetylases (HDACs) and they use β-Nicotinamide adenine dinucleotide (NAD^+^) as cofactor, different from HDAC classes I and II, which use zinc instead. They are involved in a variety of cellular functions and are regulated in response to a wide range of stimuli, including nutritional and metabolic changes, inflammatory signals and oxidative stress. Disruption of redox cellular homeostasis affects *SIRTs* at different levels, including inducing or repressing their expression, and leading to post-translational modifications such as cysteine oxidation and nitrosylation, which can lead to loss of their function (reviewed in [29]).

*SIRT1* is localized predominantly in the nucleus, but is also present in the cytosol. Among its numerous known targets are the tumor-suppressor protein p53, nuclear nactor kappa B (*NF-κB*), peroxisome proliferator-activated receptor γ coactivator 1-α (*PGC-1α*), forkhead box protein O (*FOXO*), and many other transcription factors and nuclear receptors participating in the regulation of multiple cellular functions, including mitochondrial biogenesis, glucose, and lipid metabolism, DNA repair, apoptosis, inflammation and oxidative stress resistance. In a model of atherosclerosis, lack of H_2_S-generating enzyme *CSE*, or H_2_S donor administration, have been shown to induce *SIRT1* expression and increase deacetylation activity by sulfhydration of two CXXC domains, which caused *SIRT1* to bind more zinc, therefore promoting its activity, and decreasing its ubiquitin-dependent degradation [30]. In a cardiomyocytes culture model of oxidative damage induced by hydrogen peroxide (H_2_O_2_), H9c2 cells treated with the H_2_S donor sodium hydrosulfide (NaHS) displayed a lower oxidants level and higher expression of the AOE *SOD*, *GPx* and *GST*, as well as increased *SIRT1* expression. Treatment of cells with the *SIRT1* inhibitor Ex 527 reverted the NaHS effect, indicating that H_2_S antioxidant effect was mediated through the *SIRT* pathway [31]. In an endothelial cell model of senescence induced by H_2_O_2_, treatment with NaHS resulted in increased *SIRT1* activity, although not expression, and inhibition of endothelial cell dysfunction in a *SIRT*-dependent manner [32]. Changes in *SIRT1* activity in endothelial cells after exogenous administration of NaHS have been linked to regulation of intracellular levels of NAD^+^ [33]. Diallyl trisulfide (DATS), an organosulfur compound of garlic, is a natural H_2_S donor. In a mouse model of ischemia-reperfusion injury, DATS treatment up-regulated cardiac *SIRT1* expression and nuclear distribution, leading to reduced oxidative stress and endoplasmic reticulum stress-dependent apoptosis [34].

**Figure 3:**
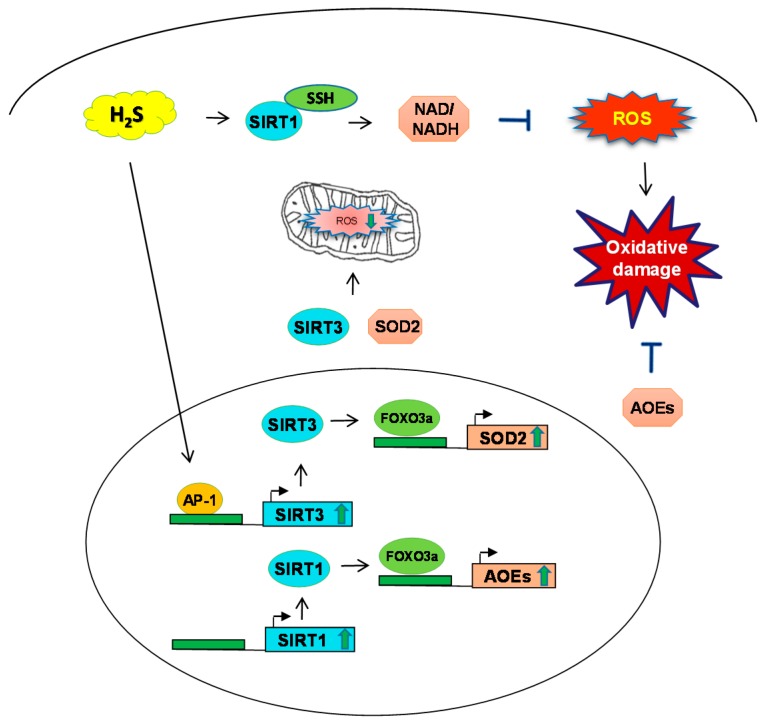
Schematic of H_2_S mechanism and sirtuins *SIRT-1*, *SIRT-3* during oxidative stress. H_2_S induces *SIRT1* to regulate the levels of nicotinamide adenine dinucleotide and nicotinamide adenine dinucleotide phosphate NAD/NADH to prevent ROS generation. *SIRT-3* induces the expression of transcription factor *FOXO3* and consequent ROS production. Additionally, H_2_S has been shown to induce *SOD2* through *SIRT3* in mitochondria and regulate oxidative stress. SSH: S-sulfhydration; AP-1: activator protein-1.

*SIRT3* is a major regulator of mitochondrial function. *SIRT3* catalyzes deacetylation of mitochondrial proteins, which in turn affects mitochondrial energy metabolism. *SIRT3* is regulated by nutritional status and metabolic stress. To investigate the ability of H_2_S to modulate oxidative stress in endothelial cells via *SIRT3* activation, the endothelial cell line EA.hy926 was pretreated with the H_2_S slow-releasing donor GYY4137, and then exposed to H_2_O_2_. GYY4137-treated cells exhibited decreased ROS formation and increased levels of total SOD activity, compared to the cells treated only with H_2_O_2_ [35]. GYY4137 treatment was able to restore the *SIRT3* expression level, which was decreased by H_2_O_2_ exposure, through increased activator protein (AP)-1 binding to the *SIRT3* promoter—effects abolished by treatment of endothelial cells with the AP-1 inhibitor SR11302 [35]. To investigate the mechanism by which H_2_S protects against cardiac hypertrophy, neonatal rat cardiomyocytes were pretreated with NaHS and treated with angiotensin II. H_2_S treatment was associated with increased *SIRT3* expression and was able to reverse angiotensin-induced mitochondrial dysfunction and *SOD2* expression (the latter was due to reduced *FOXO3a* activation—effects abolished by *SIRT3* silencing in cells [36]. In a mouse model of transverse aortic constriction (TAC) of myocardial hypertrophy, the NaHS treatment was able to reduce hypertrophy, inhibit oxidative stress, and restore mitochondria structure, volume and number only in wild-type but not *SIRT3* knockout mice [36]. The summary of the relationship between H_2_S and *SIRT* is presented in Figure 3.

## 4. H_2_S Treatment in Animal Models of Diseases Associated with Oxidative Damage

H_2_S has been recognized as playing a protective role in a variety of diseases. Reduced endogenous H_2_S levels, redox imbalance, and oxidative damage are associated with disease severity and progression in cardiac, neurological, pulmonary, gastric, nephrological, hepatic diseases, as well as in aging. H_2_S donor administration has proven beneficial in a variety of diseases associated with oxidative damage. Table 1 summarizes findings in animal models, where H_2_S donor administration results in changes in oxidative stress and/or *NRF2* activation and AOE expression/activity.

A H_2_S donor—Na_2_S—provided profound protection against myocardial ischemic injury in mice as evidenced by significant decreases in infarct size, and oxidative damage. H_2_S increased S-sulfhydration of *Keap1*, induced *NRF2* dissociation from *Keap1*, enhanced *NRF2* nuclear translocation, and expression of antioxidant enzymes to neutralize ROS [22]. Treatment with slow-releasing H_2_S donor GYY4137 protected rats against myocardial ischemia and reperfusion injury by suppressing superoxide anion levels, oxidative damage, and extracellular signal–regulated kinase ERK pathway in the myocardium [37]. H_2_S donor (NaHS) treatment in rats decreased NADPH oxidase 4-ROS-ERK1/2 signaling axis and increased heme oxygenase-1 (*HO-1*) expression and attenuated myocardial fibrotic response [38]. A novel H_2_S-donor-4-carboxyphenyl isothiocyanate (4CPI) treatment significantly decreased ROS levels, oxidative damage and ischemia/reperfusion-induced tissue injury in an in vivo model of acute myocardial infarction in rats [39]. Treatment with H_2_S donor (NaHS)-reduced NADPH oxidase 4 (NOX4) and ROS levels and cellular oxidative stress, ameliorating cardiac dysfunction in Takotsubo cardiomyopathy (TCM) in rats [40]. The organosulfur compound diallyl trisulfide (DATS) treatment in mice attenuated cardiac dysfunction after heart failure via induction of angiogenesis. DATS treatment provided a proangiogenic environment for the growth of new vessels by inducing expression of the proliferation marker, Ki67, as well as *GPx-1* and *HO-1* [41]. Treatment with NaHS significantly attenuated angiotensin II-induced hypertension and oxidative stress in mice by decreasing superoxide radical, resulting in lowered blood pressure and endothelial dysfunction [42].

H_2_S treatment offers beneficial roles in neurodegenerative disorders. Parkinson’s disease (PD) is characterized by a progressive loss of dopaminergic neurons in the substantia nigra that leads to movement dysfunction. Treatment with NaHS protected rats from 6-hydroxydopamine (6-OHDA)-induced PD by suppressing NADPH oxidase activation, ROS levels, oxidative damage, and inflammation [43]. Progressive losses of neurons and memory are hallmarks of Alzheimer’s disease (AD), and beta-amyloid plaques and oxidative stress play a crucial role in the pathogenesis. NaHS treatment in AD mice exerted antioxidant and neuroprotective effects by inducing *NRF2*, *HO-1*, *GST*, and ameliorating learning memory impairment [44]. Huntington’s disease is a fatal genetic disorder associated with accumulation of expanded polyglutamine repeats in huntingtin protein, leading to oxidative stress, neurotoxicity, and motor and behavioral changes. Recently, the researchers observed a significant depletion of *CSE*, the biosynthetic enzyme for cysteine, in Huntington’s disease tissues, and supplementation with cysteine-reverted abnormalities in a mouse model of Huntington’s disease [45].

Treatment with NaHS or GYY4137 or supplementation with L-cysteine in rats protected against gastric ischemia/reperfusion (I/R) lesions. H_2_S exerted antioxidative properties by inducing expression of *SOD2* and *GPx-1*, leading to an increase in gastric microcirculation and prevention of further progression of I/R injury into deeper gastric ulcers [46,47]. NaHS treatment in rats attenuated pulmonary I/R injury by inducing *SOD* and catalase activities, quenching superoxide production and reducing lipid damage [48]. Administration of NaHS gave protection against pulmonary fibrosis in smoking rats by attenuating oxidative stress and inflammation. H_2_S induced *NRF2* activity and up-regulated antioxidant genes *HO-1* and *Trx-1* and inhibited *NF-κB* activity in the smoking rat lungs [49]. H_2_S protected the murine liver against I/R injury through up-regulation of *GSH*, and *Trx-1* activity, attenuated lipid damage, and inhibited inflammatory factors and the progression of apoptosis [50,51]. NaHS protected rat kidneys against diabetic nephropathy and uranium-induced toxicities and murine kidneys against I/R injury through activation of the *NRF2*-antioxidant defense pathway and suppression of the inflammatory response [52,53,54].

## 5. Conclusions

An increased number of studies have confirmed the beneficial use of H_2_S donors in neuronal, cardiovascular and other oxidative stress-dependent diseases [3,4]. The role of H_2_S in modulating redox signaling has still not been fully understood, as H_2_S explicates an antioxidant effect through multiple mechanisms and interactions with different targets. Additionally, low or high cellular levels of H_2_S are linked to different outcomes of the cellular responses. The review goal was to discuss the connection between H_2_S and modulation of redox signaling and summarize the studies elucidating the role of H_2_S administration as a potential therapeutic approach for diseases due to altered redox cellular balance.

## Figures and Tables

**Table 1:** Beneficial role of H_2_S donors in animal models of oxidative stress-dependent diseases.

Model	Mechanism	H_2_S donors	Reference
**Heart**			
(Mouse)			
Ischemic heart disease	*NRF2* activation and up-regulation of AOE expression	Na_2_S	[22]
Angiogenesis	Up-regulation of AOE	DATS	[41]
Hypertension	Decrease of NADPH-dependent superoxide	NaHS	[42]
(Rat)			
Fibrosis	Decrease in ROS generation	NaHS	[38]
Myocardial ischemia	Decrease of NADPH-dependent superoxide generation	4CPI and GYY4137	[37,39]
Myocardial dysfunction	Decrease of cellular oxidative stress	NaHS	[40]
**Nervous system**			
(Mouse)			
Alzheimer’s disease	*NRF2* activation	NaHS	[44]
Huntington’s disease	Decreased oxidative stress	cysteine	[45]
(Rat)			
Parkinson’s disease	Inhibition of NADPH oxidase activity and production of ROS	NaHS	[43]
**Intestine**			
(Rat)			
Gastric ischemia-reperfusion	Up-regulation of *SOD* and *GSH-Px* activity	NaHS and GYY4137	[46]
	Decrease of free radical production	L-cysteine	[47]
**Lungs**			
(Rat)			
Ischemia–reperfusion injury	Reduction of lipid peroxidation and up-regulation of catalase, *SOD* activity	H_2_S	[48]
Pulmonary fibrosis	*NRF2* activation and up-regulation of *Trx-1*	NaHS	[49]
**Liver**			
(Mouse and Rat)			
Ischemia–reperfusion injury	Reduction of lipid peroxidation and up-regulation of	Na_2_S	[50]
	GSH and *Trx-1* activity	NaHS	[51]
**Aging**			
(Mouse)	*NRF2* activation, enhanced *SIRT1* and decreased ROS	NaHS	[26,55]
**Kidney**			
(Mouse)			
Renal Ischemia	Reduction of ROS, modulation of oxidative stress via *NRF2*	NaHS	[54]
(Rat)			
Uranium-induced toxicity	*NRF2* activation	NaHS	[52]
Diabetic nephropathy			[53]

NRF2: nuclear factor (erythroid-derived 2)-like 2; AOE: antioxidant enzyme; DATS: diallyl trisulfide; NADPH: reduced nicotinamide adenine dinucleotide phosphate; ROS: reactive oxygen species; SOD; superoxide dismutase; GSH-Px: glutathione peroxidase.
